# In-situ growth of robust superlubricated nano-skin on electrospun nanofibers for post-operative adhesion prevention

**DOI:** 10.1038/s41467-022-32804-0

**Published:** 2022-08-27

**Authors:** Yi Wang, Yuanhang Xu, Weijie Zhai, Zhinan Zhang, Yuhong Liu, Shujie Cheng, Hongyu Zhang

**Affiliations:** 1 State Key Laboratory of Tribology, Department of Mechanical Engineering, Tsinghua University, 100084 Beijing, China; 2 Basic Research Key Laboratory of General Surgery for Digital Medicine, Affiliated Hospital of Hebei University, 071000 Baoding, China; 3 State Key Laboratory of Mechanical System and Vibration, School of Mechanical Engineering, Shanghai Jiaotong University, 200240 Shanghai, China

**Keywords:** Bioinspired materials, Biomedical engineering, Biomedical materials

## Abstract

It is a great challenge to achieve robustly bonded, fully covered, and nanoscaled coating on the surface of electrospun nanofibers. Herein, we develop a controllable, facile, and versatile strategy to in-situ grow superlubricated nano-skin (SLNS) on the single electrospun nanofiber. Specifically, zwitterionic polymer chains are generated from the nanofiber subsurface in an inside-out way, which consequently form a robust network interpenetrating with the polymeric chains of the nanofiber matrix. The nanofibers with SLNS are superlubricated with the coefficient of friction (COF) lower than 0.025, which is about 16-fold of reduction than the original nanofibers. The time-COF plot is very stable after 12, 000 cycles of friction test, and no abrasion is observed. Additionally, the developed nanofibrous membranes possess favorable tensile property and biocompatibility. Furthermore, the nanofibrous membranes with SLNS achieve prevention of post-operative adhesion, which is confirmed in both rat tendon adhesion model and abdominal adhesion model. Compared with clinically-used antiadhesive membranes such as Interceed and DK-film, our nanofibrous membranes are not only more effective but also have the advantage of lower production cost. Therefore, this study demonstrates a potential of the superlubricated nanofibrous membranes in-situ grown based on a SLNS strategy for achieving prevention of post-operative adhesion in clinics.

## Abstract (web summary)

Post-operative adhesions are a major complication from surgical intervention. Here, the authors develop a technique to grow a super-lubricating layer over electrospun nanofibers which was demonstrated to prevent postoperative adhesions with better outcomes than commercial products when compared in two in vivo models.

## Introduction

Due to the highly controllable structure and performance, nanofibers produced by electrospinning have been commonly used in a wide range of areas, including energy^1^, environment^2^, and biomedical engineering^3,4^. In most of the scenarios for biomedical application, electrospun nanofibrous membranes are implanted in vivo for treatment^5^, which are in direct contact with human tissues. Therefore, the surface performance of electrospun nanofibers is crucial for their effectiveness. There have been many studies reporting about adjusting the surface properties of the electrospun nanofibers (e.g., fiber orientation^6^ and patterned structure^7^) to enhance specific cell adhesion and promote cell growth on fiber surface for the acceleration of tissue regeneration. However, excessive extrinsic adhesion and growth of cells from adjacent tissues may result in severe tissue adhesion and, accordingly, irretrievable consequences (e.g., pleuro-pericardial adhesion may lead to death)^8^. It is reported that postoperative adhesion has caused an additional medical burden of over $1 billion every year in the U.S. since the 1990s^9^. Typically, electrospun polylactic acid (PLA)-based membranes (such as DK-film) have been used in clinics for the antiadhesion purpose for many years. The membrane can form a physical barrier to separate two injured tissues, but unfortunately, it will eventually adhere to the tissue surface and thus fail to prevent the occurrence of adhesion^10,11^. Therefore, developing electrospun membranes with excellent nonadhesive surface performances is imperative and valuable in preventing postoperative adhesion in clinics.

Previously, we reported that hydration lubrication could be used to design specific nonadhesive surfaces for nanofibrous membranes^12,13^. According to the mechanism of hydration lubrication, polyelectrolyte polymers such as poly (2-methacryloyloxyethyl phosphorylcholine) (PMPC) can strongly adsorb a tenacious hydration layer around the zwitterionic charges and consequently achieve an ultralow coefficient of friction (COF) between two sliding surfaces, which prevents nonspecific cell adhesion^14^. Therefore, the key to endow electrospun membranes with nonadhesive performance without using drugs is to create a robust and consecutive zwitterionic polymer coating on the fiber surface. The greatest challenge is to optimize the interfacial bonding between the zwitterionic polymer chains in the coating and the substrate polymer chains within the nanofibers. The common method of surface modification, such as grafting polymer chains^15^ on the nanofibers, is not able to create such a robust and consecutive zwitterionic coating. This is because polymeric nanofibers such as PLA are very sensitive to organic solvents, which can dissolve PLA during surface modification. Meanwhile, the dopamine-based grafting method^16^ in an aqueous solution also cannot achieve robustly bonded and fully covered coating due to the high sensibility of dopamine to pH value and temperature in the environment.

**Fig. 1:**
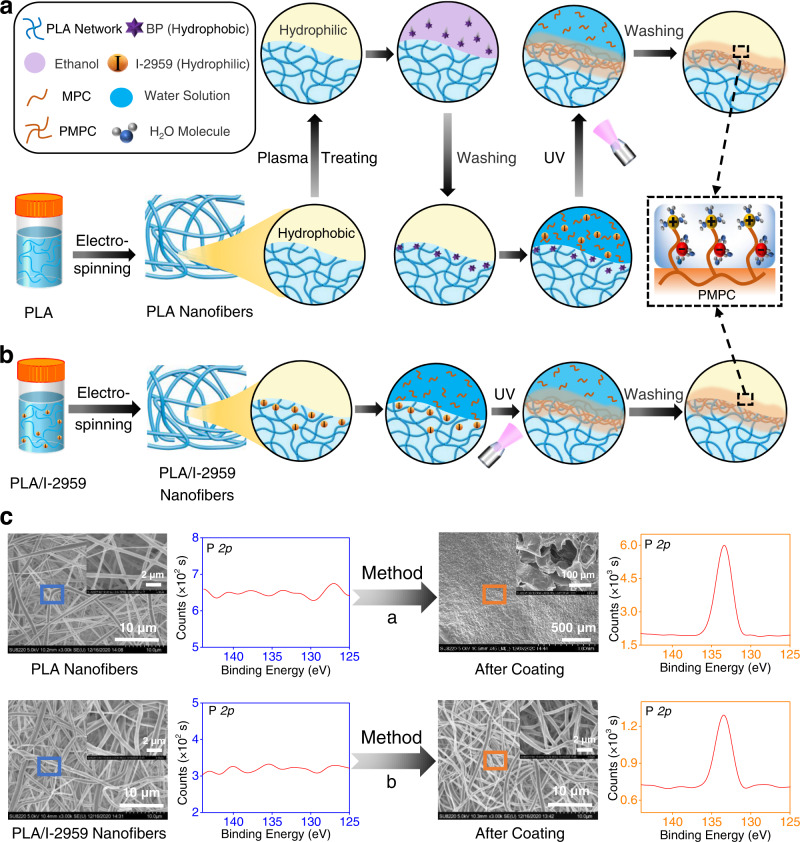
The comparison of our developed surface coating strategy to construct superlubricated nano-skin on electrospun nanofibers with previously reported method. **a** Schematic diagram of a slightly changed procedure from previous method^17,18^. PLA polylactic acid, BP benzophenone, MPC 2-methacryloyloxyethyl phosphorylcholine, PMPC poly MPC, UV ultraviolet. **b** Schematic diagram of our optimized method of subsurface-initiated polymerization. **c** Representative SEM and XPS results of electrospun nanofibers for the comparison of surface morphology and elemental composition between the method in **a** and in **b**. The experiments are replicated three times independently with similar results.

Recently, an interesting surface coating technology called “hydrogel skin” was developed, which could be widely used on diverse polymers with arbitrary shapes^17,18^. In this technique, a subsurface-initiated approach was designed to achieve outside-in growth of a hydrophilic hydrogel coating on polymeric surfaces. The special “hydrogel skin” had a typical structure of interpenetrated crosslinking with the substrate, ensuring tough and robust interfacial bonding. Two initiators with opposite hydrophilic-hydrophobic properties were applied in this method for achieving interpenetration with the substrate and “hydrogel skin” formation purposes. To examine the feasibility of this method for surface modification on electrospun nanofibers, we conducted the outside-in skin-growing procedure (using PMPC as an example) the same as previously reported (the only difference was that the substrate was replaced by electrospun PLA nanofibers), as shown in Fig. 1a. Firstly, a hydrophobic initiator benzophenone was soaked into the subsurface of plasma-treated electrospun PLA nanofibers. Afterward, the nanofibrous membranes were immersed in an aqueous solution of MPC monomer associated with a hydrophilic initiator 2-hydroxy-1-[4-(2-hydroxyethoxy)phenyl]-2-methyl-1-propanone (I-2959) and exposed under ultraviolet light for 30 min on each side. After being rinsed with sufficient deionized water to remove the weakly linked PMPC molecules, the surface-functionalized electrospun PLA nanofibers were obtained, and the presence of PMPC on the surface would endow the membranes with hydration lubrication performance.

Different from the previously reported procedure, we also developed a facile approach of subsurface-initiated inside-out growth of in situ superlubricated nano-skin (SLNS) on electrospun nanofiber surface (using PMPC as an example), as displayed in Fig. 1b. Specifically, in our approach only the hydrophilic initiator I-2959 was required. During the electrospinning process, I-2959 would further self-arrange in the subsurface within the PLA nanofibers because I-2959 was a hydrophilic small molecule and PLA was a hydrophobic polymer. It was considered that the direct electrospinning of the initiator into the subsurface could achieve an enhanced initiator concentration for further generation of in situ nano-skin, and hence there was no need of using the second initiator outside of the nanofibers. Subsequently, photopolymerization by irradiation of ultraviolet light was conducted on two sides of the electrospun PLA/I-2959 nanofibrous membranes, which were immersed in the aqueous solution of MPC monomer. Finally, the nanofibrous membranes were rinsed with sufficient deionized water to remove the unreacted MPC on the surface, and the superlubricated membranes were prepared. We compared these two different technologies by scanning electron microscopy (SEM) and X-ray photoelectron spectroscopy (XPS), as revealed in Fig. 1c. XPS is a powerful method that can investigate surface elemental compositions on the nanoscale, thus we use this technique to analyze the P element which solely originates from PMPC. From the XPS results, it can be seen that the PMPC coating has been successfully prepared using the first method. However, it is obviously shown from the SEM images that the original nanofiber structures are severely destroyed. It seems that the coating is in a microscale thickness with typical hydrogel morphologies, covering all the nanofiber regions and blocking the interconnected micropores. Therefore, the “hydrogel skin” coating is not applicable for electrospun nanofibers. In contrast, the SEM and XPS data clearly reveal that our developed technique can successfully achieve nano-skin coating without destroying the nanofiber structures. It is indicated that the in situ grown superlubricated coating is formed on the nanofiber surface with a nanoscale thickness (~1–10 nm), just like a nano-skin on every single nanofiber.

Here, we report the development of a simple yet effective way to generate superlubricated nano-skin on the surface of electrospun nanofibers. To systematically evaluate the potential of the developed electrospun nanofibrous membranes with SLNS for biomedical applications, material characterizations (such as tensile property), and a series of biological experiments (including biocompatibility and antiadhesion performance both in vitro and in vivo) are conducted. Specifically, two animal models, including the rat tendon adhesion model and abdominal adhesion model, are performed to validate the antiadhesion effectiveness. Notably, the clinically used antiadhesive membrane products, Interceed and DK-film, are set as the comparison groups in all the above-mentioned experiments.

## Results

### Material characterization

**Fig. 2:**
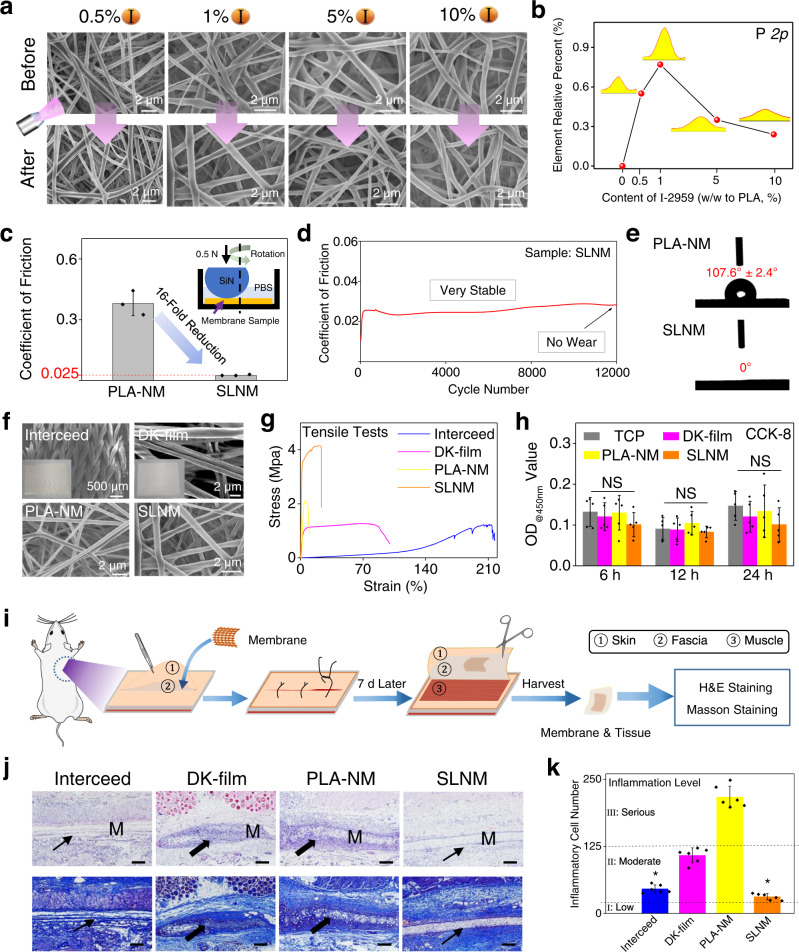
Material characterization of the superlubricated electrospun nanofibrous membranes. **a** Representative SEM images and **b** Corresponding XPS result of the nanofibrous membranes with different I-2959 contents. Scale bar: 2 µm. **c** Comparison of COF value between the PLA-NM and SLNM samples based on the tribological test operated under a rotation mode (*n* = 3 independent experiments). PLA-NM PLA nanofibrous membrane, SLNM nanofibers with superlubricated nano-skin. **d** COF-testing cycle curve of SLNM. **e** WCA result of the PLA-NM and SLNM samples. **f** Representative SEM images and **g** Tensile stress-strain curves of Interceed, DK-film, PLA-NM, and SLNM groups. **h** In vitro cell compatibility of the tested membranes (*n* = 5 independent experiments). NS no significance. **i** Schematic diagram showing the test procedure of in vivo biocompatibility evaluation. **j** Representative H&E and Masson staining images. M membrane. Scale bar: 100 µm. The black arrow points to the biofilm position, where thick arrow indicates severe inflammatory biofilm and thin arrow indicates slight inflammatory biofilm. **k** Statistical analysis of the inflammatory cell number of the Interceed, DK-film, PLA-NM, and SLNM groups (*n* = 6 independent experiments). *P = 6.56 × 10^−7^, Interceed group compared with PLA-NM group; *P = 8.36 × 10^−7^, SLNM group compared with PLA-NM group. The two-sided unpaired *t*-test is used to determine the *P*-value. The data in **c**, **h**, **k** are shown as mean ± SD, and the error bars represent SD. The experiments in **a**, **f**, **j** are replicated three times independently with similar results.

Our developed strategy for in situ growth of the superlubricated nano-skin on electrospun nanofibers is controllable, facile, and versatile. By adjusting the weight percentage of I-2959 in the electrospinning solution (from 0.5 to 10% in this study), the amount of PMPC on the surface of the nanofibers can be well controlled while the nanofiber morphology still remains the same interconnected structure (Fig. 2a). The XPS result in Fig. 2b shows that the relative PMPC intensity initially increases and then decreases with the increase in the I-2959 content, and the highest value is obtained when the I-2959 content is 1%. This phenomenon can be explained by the mechanism of free radical polymerization, which consists of chain initiation (triggered by the initiator and ultraviolet light), growth, and termination or transfer. If I-2959 concentration is low, the final PMPC chains will be very short to interpenetrate with the PLA chains, resulting in a small PMPC intensity. If I-2959 concentration is high, the cage effect will inhibit the free radical polymerization reaction, also resulting in very short PMPC chains and correspondingly small PMPC intensity. Consequently, to obtain a robust interpenetrated bonding network between the PMPC chains and PLA chains, the I-2959 concentration is crucial and should be in an appropriate range, neither too low nor too high. Thus, the I-2959 content is chosen as 1% in the electrospinning solution, and PLA/1% I-2959 is employed to prepare the superlubricated nanofibrous membranes used in the following characterizations. For short, this group of nanofibers with superlubricated nano-skin is named SLNM, while pure PLA nanofibrous membrane is named PLA-NM.

The tribological test is performed under a rotation mode to examine the lubrication performance of PLA-NM and SLNM, as shown in Fig. 2c. Interestingly, the average COF of SLNM is about 0.025, which is over 16-fold of reduction from that of PLA-NM. The surface morphology of PLA-NM and SLNM after the tribological test is presented in Supplementary Fig. 1, which indicates that the wear resistance of SLNM is better than that of PLA-NM. Additionally, the superlubricated performance of SLNM is very durable, and after 12,000 test cycles, the time-COF curve of SLNM still remains stable, and no wear is found on the surface of nanofibrous membranes (Fig. 2d). The thermogravimetric curve of PLA-NM and SLNM is shown in Supplementary Fig. 2, and the content of PMPC within SLNM is calculated to be about 5.1%. Furthermore, SLNM possesses a low static water contact angle (WCA) of almost 0° (Fig. 2e), indicating a super-hydrophilic surface of the material. However, PLA-NM is hydrophobic with a WCA value of 107.6°. Differently, in our previous work^13^, the electrospun nanofibrous membrane, which was functionalized with a dopamine-based PMPC coating, was still hydrophobic (with a WCA value over 90°) because a complete coverage of the surface coating was not achieved even at the highest grafting efficiency. Therefore, the super-hydrophilic property of SLNM indirectly indicates the presence of a fully covered and superlubricated PMPC, just like a nano-skin, on the surface of the nanofibers. Besides, to prove the universality and versatility of our developed strategy, another zwitterionic monomer, i.e., sulphobetaine methacrylate (SBMA, Supplementary Fig. 3a), is also investigated. It is shown from the XPS data (Supplementary Fig. 3b, c) that the S element is solely detected after treatment, indicating that the method is applicable for SBMA or even other similar zwitterionic monomers. More broadly, it may be appropriate for any monomers capable of free radical polymerization, such as the monomers with carbon-carbon double bonds, which can achieve specific surface functionalization of electrospun nanofibers.

To examine the potential biomedical application of SLNM, the clinically used membrane products, including Interceed (produced by Johnson & Johnson Co., U.S.) and DK-film (produced by Chengdu Dickon Pharmaceutical Co., China) as well as PLA-NM are set as the three comparison groups. The representative SEM images in Fig. 2f show that Interceed possesses a special knitted hierarchical structure based on microfibers, and it looks like a porous cloth on the macroscale. DK-film has a typical structure of electrospun microfibers, while electrospun PLA-NM and SLNM are in the nanoscale dimension (the average fiber diameters are 1.50, 0.35, and 0.36 μm for DK-film, PLA-NM, and SLNM, respectively). The result of the tribological test (Supplementary Fig. 4) shows that the average COF of Interceed and DK-film is about 0.15 and 0.39, which is higher than that of SLNM. In practical clinical applications, it is usually required for the antiadhesive membranes to be sutured with the tissues, thus an optimal mechanical performance is preferably desirable. Accordingly, the tensile tests are conducted, and the representative stress-strain curves are shown in Fig. 2g. It is indicated from the stress-strain curves that SLNM possesses the largest tensile strength of about 4 MPa, which is much higher than that of the other groups. Note that Interceed possesses the maximum breaking elongation due to its porous cloth-like structure.

Biocompatibility, also called biosafety, is a very important assessment criterion for implantable medical devices or biomaterials that are developed for clinical translation. Therefore, we evaluate the biocompatibility of the experiment group (SLNM) and all the comparison groups both in vitro and in vivo. CCK-8 measurements are conducted for in vitro cell (NIH/3T3 fibroblasts) compatibility, and the result is demonstrated in Fig. 2h. Interceed is dissolved relatively quickly in the culture medium within 12 h (Supplementary Fig. 5), as a result, the data of this group are not given. As we have noticed in our previous study^12^, fibroblasts are very weakly adhered (but not killed) to the surface of hydrophilic electrospun nanofibers, accordingly, the CCK-8 assay is performed without changing the culture medium to avoid false negative results. As expected, there are no statistical differences between SLNM and other comparison groups in all the three time points, which indicates the excellent biocompatibility of SLNM in vitro. Additionally, the in vivo biocompatibility of I-2959 is evaluated, and the result is shown in Supplementary Fig. 6. The procedure of the test for in vivo biocompatibility is illustrated in Fig. 2i, where the membranes are subcutaneously implanted between the skin and the fascia. The hematoxylin-eosin (H&E) and Masson staining result is shown in Fig. 2j, and the corresponding semi-quantitative analysis of the inflammatory cells is revealed in Fig. 2k. It is clear from these staining images that SLNM and Interceed possess optimal biocompatibility with very little inflammatory biofilm invasion, but PLA-NM and DK-film show obvious sites for severe inflammatory biofilm invasion. Consistently with the above staining result, the inflammatory cell number of the SLNM and Interceed groups is significantly lower (*p* < 0.05) than that of the PLA-NM group and much smaller than that of the DK-film group. The inflammation level of SLNM is very close to level I (i.e., little inflammation), which demonstrates the optimal biosafety of SLNM compared with the other materials. Taking all the results into consideration, the developed SLNM possesses excellent biocompatibility both in vitro and in vivo.

### In vitro and in vivo antiadhesion properties

**Fig. 3:**
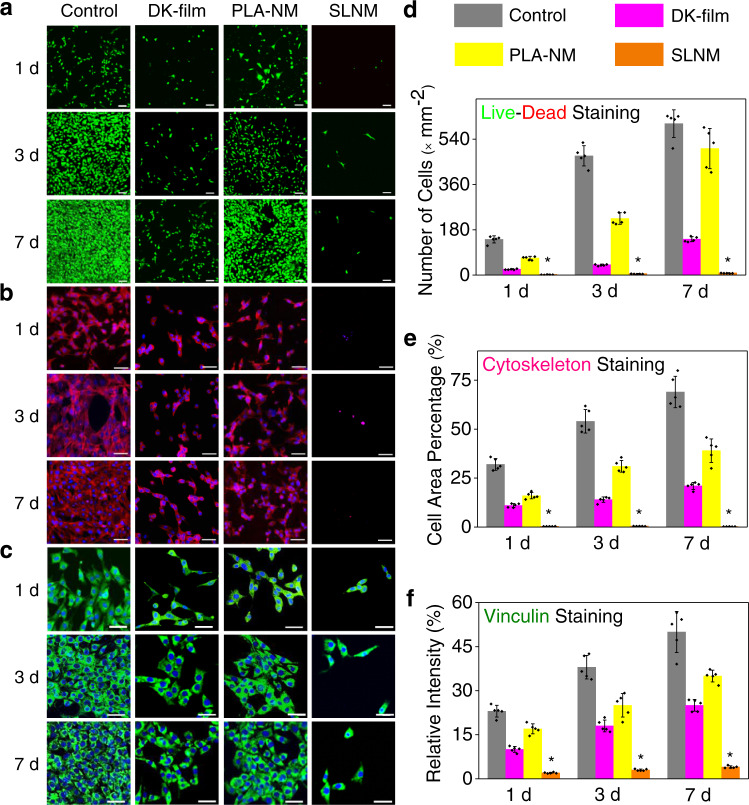
In vitro anticell adhesion properties of the superlubricated electrospun nanofibrous membranes. Confocal laser scanning microscopic images for **a** Live/dead staining (scale bar: 100 µm), **b** Cytoskeleton staining (scale bar: 50 µm), and **c** Vinculin staining (scale bar: 50 µm) of NIH/3T3 fibroblasts co-cultured with DK-film, PLA-NM, and SLNM on 1, 3, and 7 d, respectively. Statistical analysis of the corresponding semi-quantitative data of the **d** Adhered cell number, **e** Cell area percentage, and **f** Relative vinculin expression for the DK-film, PLA-NM, and SLNM groups (*n* = 5 independent experiments). **d** *P_1d_ = 3.60 × 10^−6^, compared with DK-film group; *P_1d_ = 2.71 × 10^−5^, compared with PLA-NM group. *P_3d_ = 5.57 × 10^−6^, compared with DK-film group; *P_3d_ = 8.01 × 10^−3^, compared with PLA-NM group. *P_7d_ = 6.73 × 10^−6^, compared with DK-film group; *P_7d_ = 1.60 × 10^−4^, compared with PLA-NM group. **e** *P_1d_ = 1.73 × 10^−5^, compared with DK-film group; *P_1d_ = 2.64 × 10^−5^, compared with PLA-NM group. *P_3d_ = 2.35 × 10^−8^, compared with DK-film group; *P_3d_ = 2.26 × 10^−5^, compared with PLA-NM group. *P_7d_ = 1.35 × 10^−5^, compared with DK-film group; *P_7d_ = 1.32 × 10^−4^, compared with PLA-NM group. **f** *P_1d_ = 1.12 × 10^−7^, compared with DK-film group; *P_1d_ = 4.70 × 10^−8^, compared with PLA-NM group. *P_3d_ = 5.73 × 10^−5^, compared with DK-film group; *P_3d_ = 2.39 × 10^−4^, compared with PLA-NM group. *P_7d_ = 1.11 × 10^−5^, compared with DK-film group; *P_7d_ = 6.10 × 10^−10^, compared with PLA-NM group. The two-sided unpaired *t*-test or Mann–Whitney *U* test is used to determine the *P* value. The data in **d**–**f** are shown as mean ± SD, and the error bars represent SD. The experiments in **a**–**c** are replicated three times independently with similar results.

Initially, NIH/3T3 fibroblasts are seeded on the surface of the tested membranes to evaluate their antiadhesive performance in vitro. The results of the live/dead staining, cytoskeleton staining, and vinculin staining of the cells co-cultured with the materials on 1, 3, and 7 d are shown in Fig. 3. Obviously, the adhered cell number (Fig. 3a, d), cell area percentage (Fig. 3b, e), and relative vinculin expression (Fig. 3c, f) of the SLNM group are all significantly reduced compared with the other three groups, with a statistical difference of *p* < 0.05. Additionally, the anticell adhesion behavior of the membrane is not affected by extending the culture time from 1 day to 7 days, indicating that SLNM possesses a very stable and durable antiadhesive property in vitro. Comparing the SLNM group with the PLA-NM and DK-film groups, the main difference is that SLNM is endowed with a superlubricated nano-skin on the surface. Therefore, it can be concluded from the in vitro experiment that the excellent and long-lasting anticell adhesion performance of SLNM is highly associated with its superlubricated surface.

**Fig. 4:**
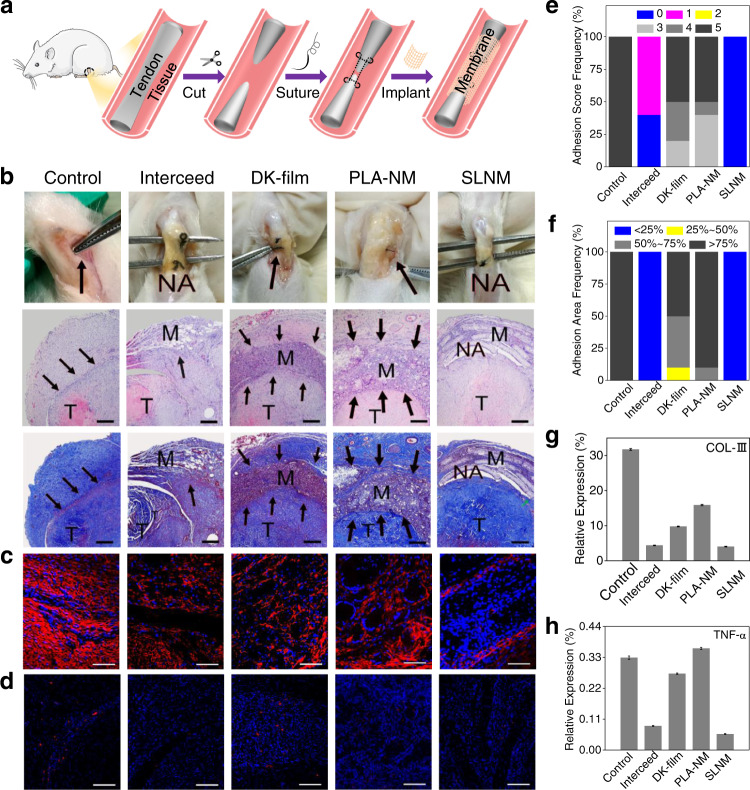
In vivo antitissue adhesion properties of the superlubricated electrospun nanofibrous membranes based on rat tendon adhesion model. **a** Schematic diagram showing the overall animal test process. **b** Photos of the harvested tendon on 14 d following implantation and H&E staining as well as Masson staining images. Scale bar: 500 µm. The black arrows point to adhesion site. M membrane. T tendon. NA no adhesion. Representative confocal laser scanning microscopic images for the immunofluorescent staining of **c** COL-III (scale bar: 100 µm) and **d** TNF-α (scale bar: 200 µm). Red color represents targeted protein and blue color represents cell nucleus. Comparison of **e** Adhesion score, **f** Adhesion area, and relative expression levels of **g** COL-III as well as **h** TNF-α for the control, Interceed, DK-film, PLA-NM, and SLNM groups, respectively. The experiments in **b**–**d** are replicated three times independently with similar results.

To further investigate the potential of the superlubricated electrospun nanofibrous membranes for preventing postoperative adhesion in clinical applications, two typical animal models, including the rat tendon adhesion model and abdominal adhesion model, are performed. Figure 4 displays the results of the rat tendon adhesion experiment, and the schematic diagram in Fig. 4a shows the establishment of the rat tendon adhesion model. Specifically, the rat tendons are initially transected in the middle using scissors and then sutured (Supplementary Fig. 7). Subsequently, the membrane samples are sterilized and implanted, covering the tendons (Supplementary Fig. 8). Finally, the membrane samples are fixed by suturing, and the rat skins are closed. The tendon-material composites are harvested at predetermined time. As shown in Supplementary Fig. 9, the tissue harvesting time is set as 14 days following material implantation due to the occurrence of severe adhesion. It is clear from the photos of the harvested tissues that the Interceed and SLNM groups possess excellent antiadhesive performance, as the surgical scissors can easily cross the area between the tendon and the underlying fibrous tissues (Fig. 4b). However, it is very difficult for the surgical scissors to pass through in all the other comparison groups, and it indicates the occurrence of severe adhesion at the tendon-fibrous tissue interface. The analysis of the adhesion score in Fig. 4e shows that all the SLNM samples obtain zero score, which represents the complete prevention of tendon adhesion. By contrast, the other comparison groups exhibit tendon adhesion with different adhesion scores. Additionally, the H&E staining and Masson staining are conducted to observe tissue adhesion in the cell-scale situation, as illustrated in Fig. 4b. Similarly, no adhesion is detected in the SLNM group, while limited biofilm formation is found in the Interceed group. However, there is severe and excessive fibrosis for the samples in the control, DK-film, and PLA-NM groups. Consistently, the analysis of the adhesion area (Fig. 4f), which is calculated based on the staining images, shows that the SLNM and Interceed samples all possess an adhesion area of lower than 25%, indicating excellent antiadhesion performance. Furthermore, the immunofluorescent staining of two typical proteins (i.e., COL-III and TNF-α), which are involved in the process of adhesion formation, is also performed in order to verify the above results. As expected, the representative confocal laser scanning microscopic images (Fig. 4c, d) and the corresponding semi-quantitative results (Fig. 4g, h) illustrates that the samples in the Interceed and SLNM groups exhibit significantly lower expression levels of COL-III and TNF-α compared with the other three groups, indicating excellent antitissue adhesion performance.

**Fig. 5:**
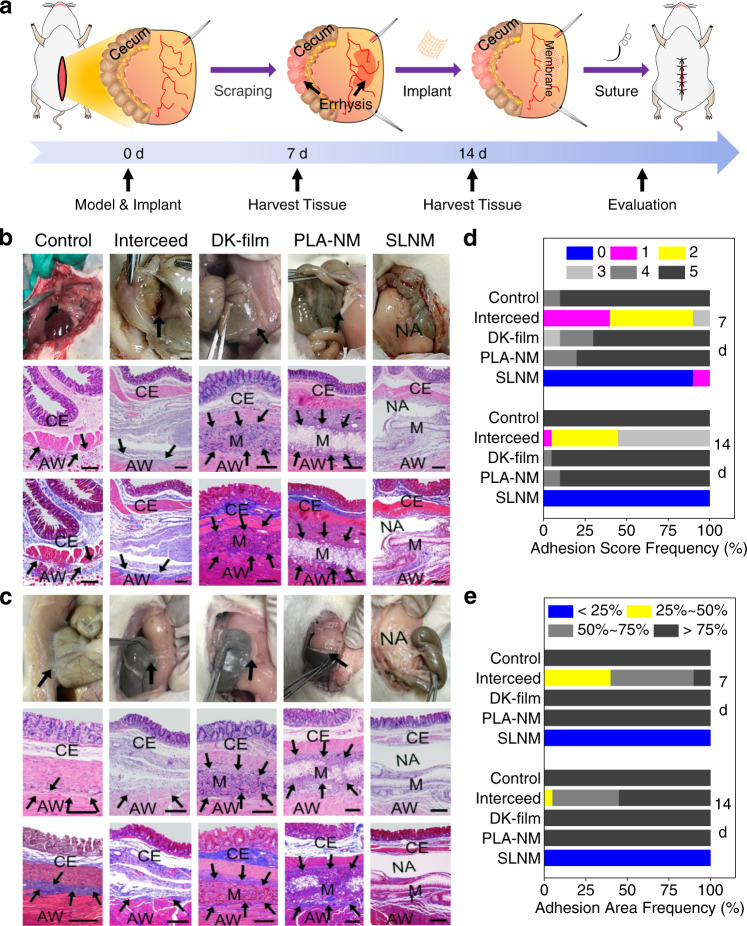
In vivo antitissue adhesion properties of the superlubricated electrospun nanofibrous membranes based on rat abdominal adhesion model, which is achieved by scraping the abdominal wall surface until errhysis. **a** Schematic diagram showing the overall animal test process. Photos of the harvested cecum and abdominal wall and H&E staining as well as Masson staining images of the harvested tissues on **b** 7 d and **c** 14 d. Scale bar: 200 µm. The black arrows point to adhesion site. CE cecum. M membrane. AW abdominal wall. NA no adhesion. Comparison of **d** Adhesion score and **e** Adhesion area for the control, Interceed, DK-film, PLA-NM, and SLNM groups on 7 and 14 d, respectively. The experiments in **b**, **c** are replicated three times independently with similar results.

The results of the rat abdominal adhesion experiment are demonstrated in Fig. 5, and the schematic diagram in Fig. 5a shows the establishment of the rat abdominal adhesion model, which is achieved by scraping the surface of the abdominal wall until the occurrence of errhysis is observed (Supplementary Fig. 10). Afterward, the membrane samples are implanted to separate the cecum and opposite abdominal wall, and then fastened on the surface of abdominal wall by suturing before skin closure (Supplementary Fig. 11). In this test, the tissue harvesting time is set as 7 and 14 days following material implantation as on both time points severe tissue adhesion is detected, as shown in Supplementary Fig. 12. Interestingly, the SLNM group still shows perfect prevention of tissue adhesion, as revealed from the photos (Fig. 5b, c corresponding to 7 d and 14 d) and adhesion score (Fig. 5d). On 7 d, it is clear that the cecum completely separates from partly-degraded SLNM, and more than 90% of the samples in the SLNM group possess zero score. On 14 d, all the samples in the SLNM group obtain zero score, and retain the complete antiadhesion performance between the cecum and abdominal wall during membrane degradation. By contrast, the other comparison groups all display tissue adhesion to different degrees. For example, even for the well-known clinical product, Interceed, there are still obvious adhesion sites, and the adhesion scores are ranged between 1 and 3 on 7 d and 14 d. In addition, the H&E staining and Masson staining images in Fig. 5b, c associated with the analysis of the adhesion area in Fig. 5e are highly consistent with the above-mentioned observations, indicating that the antiadhesion performance of the samples in the SLNM group is the best among the different materials tested. Taken all the results of two animal models together, it is concluded that the developed superlubricated electrospun nanofibrous membranes are endowed with excellent performance for preventing postoperative tissue adhesion.

### Antiadhesion mechanism

**Fig. 6:**
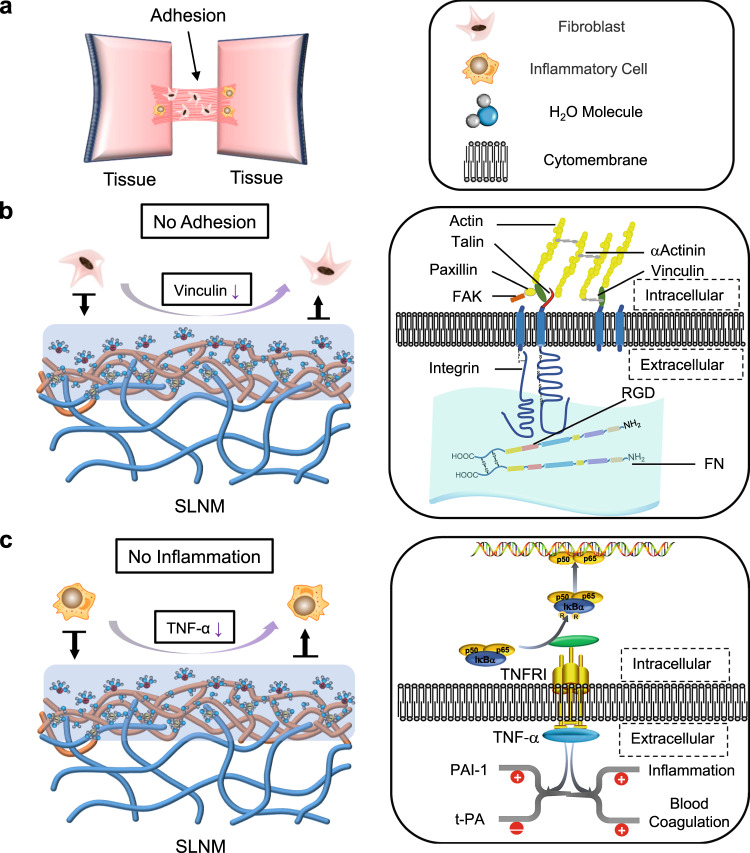
Potential antiadhesion mechanism of the superlubricated electrospun nanofibrous membranes. **a** Schematic diagram showing the occurrence of postoperative tissue adhesion. Interstitial fibrosis and inflammation are involved in this process. Schematic diagrams showing the mechanisms of **b** inhibiting fibrosis and **c** reducing inflammation based on the tenacious hydration layer formed surrounding the zwitterionic phosphorylcholine groups on the SLNM surface.

The potential mechanism for the excellent antiadhesion performance of SLNM is shown in Fig. 6. Generally, it is accepted that the formation of tissue adhesion mainly involves interstitial fibrosis and inflammation activation (Fig. 6a)^19,20^. During the process of fibrosis, the adhesion of fibroblasts to the surface of the material is the key factor. Fibronectin (FN), which contains arginine-glycine-aspartic (RGD) peptide sequence, is usually distributed on the cell membrane, and integrin is the receptor of FN (Fig. 6b)^21,22^. Vinculin is mediated by the combination of FN and integrin, and it is crucial for cell adhesion^23,24^. At the beginning of the fibroblast-material interactions, it is difficult for FN to be adhered to the SLNM surface because of the tenacious hydration layer formed surrounding the zwitterionic phosphorylcholine groups, which can produce a hydration repulsion effect on the approaching molecules (Fig. 6b). Consequently, FN in the fibroblasts and integrin on the SLNM surface cannot be combined, and the expression level of vinculin is sharply reduced to almost zero, resulting in the excellent property for preventing the adhesion of fibroblasts. On the other hand, the inflammatory factor, TNF-α, is also important for triggering a series of consecutive immune responses that can mediate the development of tissue adhesion (Fig. 6c)^25^. Similarly, the tenacious hydration layer that fully covers the SLNM can completely inhibit the adhesion of the inflammatory cells on the surface, and correspondingly the expression level of TNF-α is greatly reduced, and no inflammation is observed (Fig. 6c). Actually, the reduction in the expression levels of COL-III and TNF-α has been proved by immunofluorescent staining in the rat tendon adhesion test. Accordingly, there are almost no adhesion and no inflammation for the developed superlubricated electrospun nanofibrous membranes, indicating an excellent performance for the prevention of postoperative tissue adhesion.

**Fig. 7:**
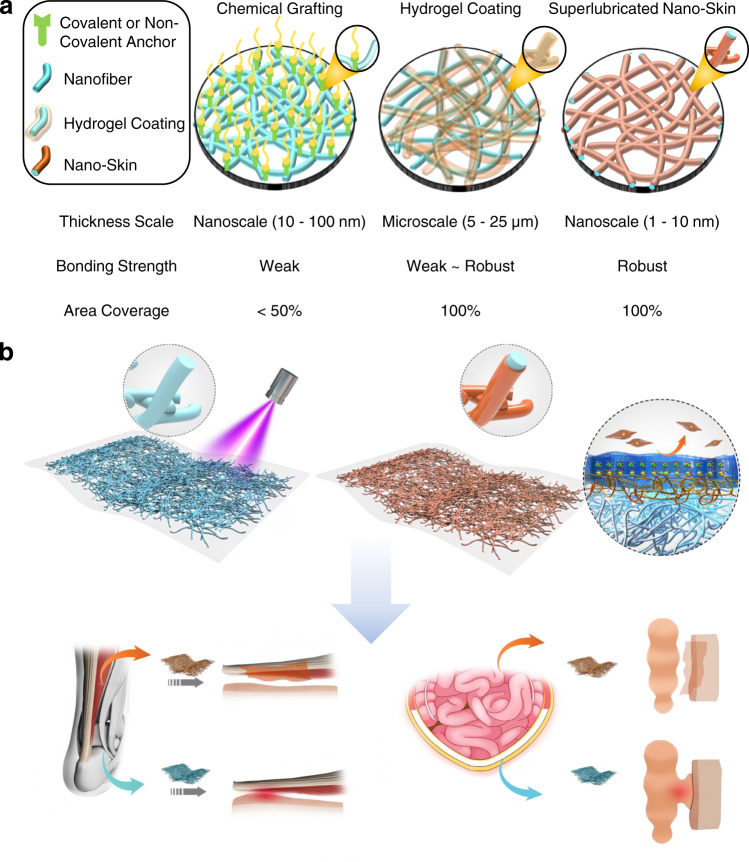
Superlubricated nano-skin on electrospun nanofibers for postoperative adhesion prevention. **a** Comparison among three representative surface modification methods on electrospun nanofibers including chemical grafting, hydrogel coating, and superlubricated nano-skin coating. **b** A subsurface-initiated photopolymerization method is developed to achieve in situ growth of robust and superlubricated nano-skin on electrospun nanofibers. The superlubricated nanofibrous membranes are endowed with excellent performances of preventing postoperative adhesion, which are validated by in vivo antitissue adhesion tests based on rat tendon adhesion model and abdominal adhesion model.

Figure 7a displays representative surface functionalization technologies which may be potentially applied for electrospun nanofibers. Surface chemical grafting^15,16,26–31^ is the most commonly used method, such as dopamine-based grafting, cyclodextrin-based supramolecular grafting, etc. However, there are obvious limitations to this technique. For example, dopamine is sensitive to ambient temperature and pH values which can affect the bonding strength. Supramolecular grafting^27^, a method proposed based on the noncovalent interactions, is good at dynamic self-healing but inherently unstable. Therefore, it is considered that this method cannot produce a robust functionalized coating on the surface of electrospun nanofibers. It is indicated that both the lubrication property and the antiadhesion performance of surface chemical grafting were inferior to the SLNS strategy developed in this study^13^. Very recently, hydrogel coating has been developed on diverse polymeric materials with a flexible and biocompatible performance^17,18,32–37^. Typically, we followed a similar procedure of this technique to prepare a hydrogel coating on electrospun nanofibers, as shown in Fig. 1a. Unfortunately, the hydrogel coating, which was generated on the surface in an outside-in manner, was on a microscale and thus destroyed the original interconnected porous structure of the nanofibers. In addition, it is indicated from the in vivo biocompatibility test of the membrane modified by hydrogel coating that a slightly moderate degree of inflammation is observed in the H&E and Masson staining images, as shown in Supplementary Fig. 13a. The average COF of the membrane is about 0.30 (Supplementary Fig. 13b, c), which is much higher (12-fold) than that obtained by the SLNS strategy (0.025). This result may be related to the visible and macroscopic hydrogel coating on the nanofibrous membrane surface, which is not resistant to abrasion due to reduced bonding strength. Consequently, the SLNS strategy has an obvious advantage over hydrogel coating in the aspect of in vivo biocompatibility and lubrication property. As a proof-of-concept, the subsurface-initiated photopolymerization approach (Figs. 1b, 7b) developed herein achieved in situ inside-out growth of robust and fully covered superlubricated nano-skin on every single electrospun nanofiber. The interpenetrated architecture of the polymeric chains ensured that the superlubricated nano-skin on the electrospun nanofibers was stable and durable. Based on the results of the present study and our previous research^12,13^, the potential threshold for COF of the nanofibrous membrane in antiperitoneal adhesion was considered to be 0.12–0.15.

## Discussion

In this study, we developed a controllable, facile, and versatile strategy to achieve in situ growth of superlubricated nano-skin on every single electrospun nanofiber. This special functionalized coating was based on subsurface-initiated photopolymerization, and could be produced from any zwitterionic monomers. The superlubricated nano-skin was robust and fully covered on the electrospun nanofibrous membranes because of the interpenetrated architecture of the polymeric chains between the coating and nanofiber matrix. Most interestingly, the developed SLNM with the superlubricated nano-skin showed significantly better antiadhesion performance compared to two clinically used products (Interceed and DK-film), which was validated by the in vitro anticell adhesion test and in vivo antitissue adhesion test from rat tendon adhesion model and abdominal adhesion model. In conclusion, it is anticipated that the unique technique for preparing superlubricated nano-skin on electrospun nanofibrous membranes will open up exciting horizons for the design and development of superlubricated biomaterials for the purpose of preventing postoperative adhesion.

## Methods

### Material preparation

PLA (PDLLA, average molecular weight: 86, 000), I-2959, and other chemicals were purchased from J&K Scientific Co., Beijing, China. A hexafluoro-isopropanol (HFIP) solution of the PLA (15%, w/v to HFIP)/I-2959 (0.5–10%, w/w to PLA) composites was electrospun into isotropic nanofibrous membranes employing the following parameters: voltage 20 kV, feeding speed 6 mL/h, collecting distance 18 cm, and room temperature. Subsequently, the nanofibrous membranes were dried in a vacuum chamber for 3 days to remove the remaining solvents. To prepare SLNS on the surface of the PLA/I-2959 nanofibers, the samples were immersed in a saturated MPC (10%, w/v) aqueous solution and then exposed under ultraviolet light for 40 min/side using an OmniCure S2000 lamp at an intensity of 100 mW/cm^2^ (measured at 365 nm). After the reaction, the nanofibrous membranes were rinsed with sufficient deionized water at least 10 times, naturally dried, and finally stored under vacuum until use. The procedure displayed in Fig. 1a was described in Introduction section for the purpose of comparison.

### Material characterization

The membrane samples were characterized by SEM and XPS for surface morphology and elemental composition analysis. For SEM observation, the samples were sprayed with a layer of Pt by a vacuum ion sputtering apparatus (EM ACE600, Leica, Germany) for 10 min, and then examined utilizing a scanning electron microscope (SEM, Quanta200, FEI, Eindhoven, Netherlands) at random views. The images were acquired using Thermo Scientific Maps software (version 3.21). For the elemental composition analysis, the samples were evaluated by an X-ray photoelectron spectrometer (XPS, ESCALAB 250XI, Thermo Fisher, Waltham, USA). The data were collected and analyzed using Avantage software (version 5.9918). Additionally, the lubrication performance, component content, surface wettability, and mechanical property of the membranes were characterized based on a series of tribological tests, thermogravimetric analysis, WCA measurement, and tensile test, respectively. The tribological test was performed using a universal materials tester (UMT-5, Bruker Nano Inc., Germany) under a rotation mode (rotation speed: 50 mm min^−1^, normal load: 0.5 N, rotation radius: 3 mm, lubricant medium: deionized water). The membrane samples were used as the lower specimens, which were firmly bonded to the top surface of the glass slide using 3 M tape and slid against the upper specimens, i.e., SiN balls with a diameter of 5 mm. The thermogravimetric curve of the membranes was obtained using a thermal analysis coupled system (X70, Netzsch group, Selb, Germany) at the heating rate of 10 °C/min over a temperature range of 20–1000 °C under a constant stream of nitrogen (20 mL/min). The WCA value of the membranes was measured using a goniometer (OCA-20, Dataphysics Instruments, Filderstadt, Germany) based on the sessile drop method by dropping 5 μL of deionized water on the sample surface. The tensile strength of the membrane samples (size: 5 cm × 6 mm) was examined using a material testing machine (Instron 5567, Norwood, MA, USA) at room temperature. The data were collected using Instron Bluehill software (version 3.0).

### Biocompatibility evaluation

The biocompatibility of the membranes was evaluated both in vitro and in vivo. For the in vitro test, NIH/3T3 fibroblasts were seeded on the surface of the membranes, and the Cell Counting Kit-8 (CCK-8) assay was performed after incubation of 6, 12, and 24 h without exchange of culture media, respectively. The absorbance of the solution was recorded using a microplate reader (Varioskan Flash, Thermo Fisher, Waltham, USA) at a wavelength of 450 nm. Regarding the in vivo test, the membranes were subcutaneously implanted into the rat back between the skin and the fascia. The material-tissue composites were harvested on 7 d, and H&E staining, as well as Masson staining, were conducted. The images were obtained using a microscope (Olympus BX53, Japan) with Olympus Image Viewer software (cellSens standard, version 3.1). The inflammatory cell number was calculated by using the ImageJ software (version 1.53q) for comparison based on the staining images.

### In vitro test of anticell adhesion

NIH/3T3 fibroblasts were used to evaluate the in vitro anticell adhesion property of the membranes. Specifically, the membrane samples with a diameter of 15 mm were placed in 24-well culture plates and sterilized using ultraviolet light for 24 h. Subsequently, the cell suspension with a density of 4 × 10^4^ per well was seeded on the surface of the samples and incubated at 37 °C and 5% CO_2_. The live/dead staining, cytoskeleton staining, and vinculin staining were performed and then observed using a confocal laser scanning microscope (LSM-800, ZEISS, Germany) on 1, 3, and 7 d, respectively. The data were collected by ZEN software (version 2.3). Rabbit Anti-Vinculin Monoclonal Antibody (Abcam, ab129002, Monoclonal, 1:200) was used for vinculin staining. The semi-quantitative data, including adhered cell number, cell area percentage, and relative vinculin expression, were obtained utilizing the ImageJ software (version 1.53q) based on these images.

### In vivo test of antitissue adhesion

Two animal models were used to investigate the in vivo antitissue adhesion properties of the membranes, including rat tendon adhesion model and the abdominal adhesion model. Briefly, male SD rats (body weight: 200 ± 25 g) were purchased from SPF (Beijing) Biotechnology Co., Ltd, China. The procedures for performing the animal test were approved by the Ethics Committee of Hebei University. During the tests, the International Ethical Guidelines and National Institutes of Health Guide concerning the Care and Use of Laboratory Animals were strictly followed. The rats were randomly divided into five groups (six rats in each group), including the control (adhesion without any treatment), Interceed, DK-film, PLA-NM, and SLNM groups.

### Rat tendon adhesion model

The SD rats were anesthetized with chloral hydrate (5%, w/v to saline), and shaved for the heels of the right hind before surgery. Initially, a posterior midline incision was made in the right posterior heel, and afterward, the tendon sheath was dissected longitudinally. The tendons were isolated and transected at the midpoint of the muscle-tendon transition and calcaneal insertion. Subsequently, the modified Kessler method was used to suture the ruptured tendon, and then the tendons were covered by the membrane samples. After surgery, the rats were sacrificed at a predetermined time (14 days following implantation in this test), and the tissue-membrane composites were harvested and evaluated based on gross observation, adhesion score, H&E staining, and Masson staining. The adhesion score was determined by the same criteria as reported in our previous study^12^. Additionally, the immunofluorescent staining of COL-III and TNF-α was performed and observed using the confocal laser scanning microscope. The following primary and secondary antibodies were used in this study, including Rabbit Anti-TNF alpha Polyclonal Antibody (Bioss, bs-10802R, Polyclonal, 1:200), Rabbit Anti-Collagen III Polyclonal Antibody (Servicebio, GB111629, Polyclonal, 1:500), and Goat Anti-Rabbit IgG (H + L) Highly Cross-Adsorbed Secondary Antibody (Invitrogen, A11034, 1:400). The semi-quantitative data including the adhesion area and the relative expression levels of COL-III and TNF-α were examined by the ImageJ software (version 1.53q).

### Rat abdominal adhesion model

In the rat abdominal adhesion model, the cecum and opposite abdominal wall were exposed and scraped with sterile surgical gauze until the occurrence of errhysis. Subsequently, the membrane samples with an area of 1 × 1 cm^2^ were implanted to cover the injured site and sutured to the abdominal wall for fixation. The cecum was placed back into the abdominal cavity and fixed against the injured site of the abdominal wall. Finally, the incision was sutured layer by layer to close the abdomen. After surgery, the rats were sacrificed at a predetermined time (7 and 14 days following implantation in this test), and similarly, the tissue-membrane composites were harvested and evaluated based on gross observation, adhesion score, H&E staining, and Masson staining. The semi-quantitative data of the adhesion area were also obtained employing the ImageJ software (version 1.53q).

### Statistical analysis

The quantitative data were shown as mean ± standard deviation (SD), and the Shapiro-Wilk test was employed to verify the normality of the data. When the data were normally distributed, the two-sided unpaired *t*-test was used to determine the statistical significance between two test groups, otherwise, the Mann–Whitney *U* test was used to determine the statistical significance. The statistical analysis was performed using the Statistical Product and Service Solutions (SPSS) software (version 19.0). The statistical difference was considered significant when *p < 0.05.

### Reporting summary

Further information on research design is available in the Nature Research Reporting Summary linked to this article.

## Supplementary information

Supplementary Information

Reporting Summary

## Data Availability

All the data supporting the findings of this study are available within the main text of this article and its Supplementary Information, or from the corresponding authors upon request. Source data are provided with this paper.

## Source data

Source Data
