# Supplementary material for: In-situ growth of robust superlubricated nano-skin on electrospun nanofibers for post-operative adhesion prevention
Source: Nat Commun. 2022 Aug 27;13:5056. doi: 10.1038/s41467-022-32804-0 (PMC9420117; doi:10.1038/s41467-022-32804-0)
Supplement: Supplementary file 1 — Supplementary Information [file 41467_2022_32804_MOESM1_ESM.pdf]

Supplementary Information

In-situ growth of robust superlubricated nano-skin on electrospun nanofibers for post-operative adhesion prevention

Yi Wang[1, 2], Yuanhang Xu[2], Weijie Zhai[2], Zhinan Zhang[3], Yuhong Liu[1], Shujie Cheng[2, *], and Hongyu Zhang[1, *]

[1] State Key Laboratory of Tribology, Department of Mechanical Engineering, Tsinghua University, Beijing 100084, China

[2] Basic Research Key Laboratory of General Surgery for Digital Medicine, Affiliated Hospital of Hebei University, Baoding 071000, China

[3] State Key Laboratory of Mechanical System and Vibration, School of Mechanical Engineering, Shanghai Jiaotong University, Shanghai 200240, China

[*] Corresponding Authors

Prof. Cheng S.J. (E-mail: chengshuj@126.com)

Prof. Zhang H.Y. (E-mail: zhanghyu@tsinghua.edu.cn)

Table of Contents

Supplementary Fig.1 The evaluation of surface morphology of nanofibrous membrane following the tribological test. **a** PLA-NM. **b** SLNM. Scale bar: 2 mm.

Supplementary Fig.2 The thermogravimetric analysis (TGA) of PLA-NM and SLNM samples.

Supplementary Fig.3 Development of superlubricated nano-skin on electrospun nanofibers using the zwitterionic monomer SBMA as another example. **a** Structure of PLA and SBMA. **b** XPS curve of electrospun nanofibers before surface coating. **c** XPS curve of electrospun nanofibers after surface coating.

Supplementary Fig.4 The result of the tribological test operated under a rotation mode. **a** COF-testing cycle curve of Interceed in air. **b** COF-testing cycle curve of Interceed in water. Scale bar: 7.5 mm. Note that a larger rotation radius was used in the tribological test to fulfil the experiment under an aqueous condition. **c** COF-testing cycle curve of DK-film in air. **d** Comparison of COF value between Interceed and DK-film. The data in **d** are shown as mean ± SD, and the error bars represent SD (n = 3 independent experiments).

Supplementary Fig.5 Photos of Interceed membrane after being soaked in the culture medium within 12 h.

Supplementary Fig.6 The *in vitro* biocompatibility evaluation of I-2959 based on CCK-8 assay by co-culturing with fibroblasts for 24 h. **a** Optical density (OD) values of control and I-2959 groups at 450 nm. **b** Relative biocompatibility calculated from the OD values. The data in **a** and **b** are shown as mean ± SD, and the error bars represent SD (n = 4 independent experiments).

Supplementary Fig.7 Photos showing the typical procedures for establishing rat tendon adhesion model. **a** anesthetization. **b** tendon exposure. **c** pre-immobilization. **d** tendon transection. **e** suturing. **f** skin closure.

Supplementary Fig.8 Photos showing the implantation of the membrane samples in the Interceed, DK-film, PLA-NM, and SLNM groups before skin closure in the rat tendon adhesion model.

Supplementary Fig.9 A preliminary test for the determination of tissue harvesting time in the rat tendon adhesion model.

Supplementary Fig.10 Photos showing typical procedures for establishing rat abdominal adhesion model. **a** anesthetization. **b** abdomen incision exposing cecum and

opposite abdominal wall. **c** cecum errhysis. **d** abdominal wall errhysis before skin closure.

Supplementary Fig.11 Photos showing the implantation of the membrane samples in the Interceed, DK-film, PLA-NM, and SLNM groups before skin closure in the rat abdominal adhesion model. The membranes were sutured on the surface of the abdominal wall.

Supplementary Fig.12 A preliminary test for the determination of tissue harvesting time in the rat abdominal adhesion model.

Supplementary Fig.13 The biocompatibility and lubrication properties of nanofibrous membrane modified by hydrogel coating. **a** Representative H&E and Masson staining images. M: Membrane. Scale bar: 200 μm. Red arrow points to the inflammation position. The experiments were replicated three times independently with similar results. **b** COF-testing cycle curve in air. **c** COF-testing cycle curve in water.

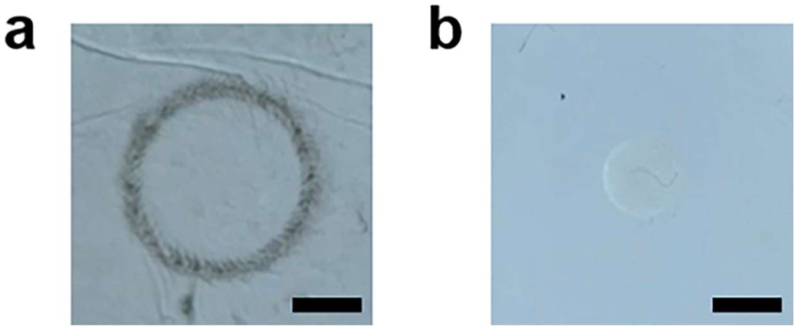

Supplementary Fig.1 The evaluation of surface morphology of nanofibrous membrane following the tribological test. **a** PLA-NM. **b** SLNM. Scale bar: 2 mm.

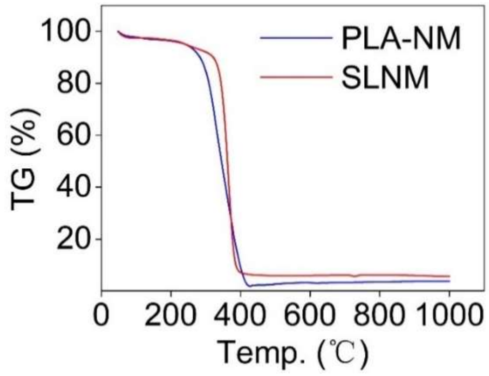

Supplementary Fig.2 The thermogravimetric analysis (TGA) of PLA-NM and SLNM samples.

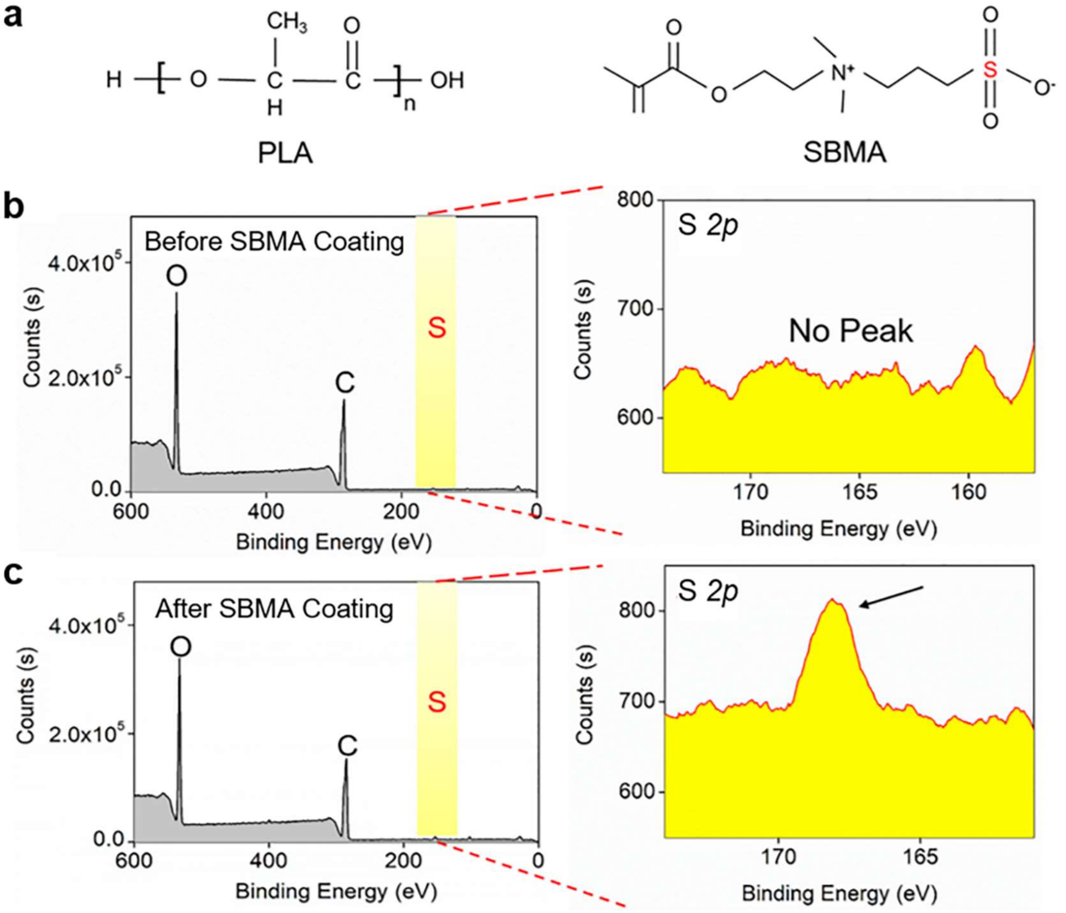

Supplementary Fig.3 Development of superlubricated nano-skin on electrospun nanofibers using the zwitterionic monomer SBMA as another example. **a** Structure of PLA and SBMA. **b** XPS curve of electrospun nanofibers before surface coating. **c** XPS curve of electrospun nanofibers after surface coating.

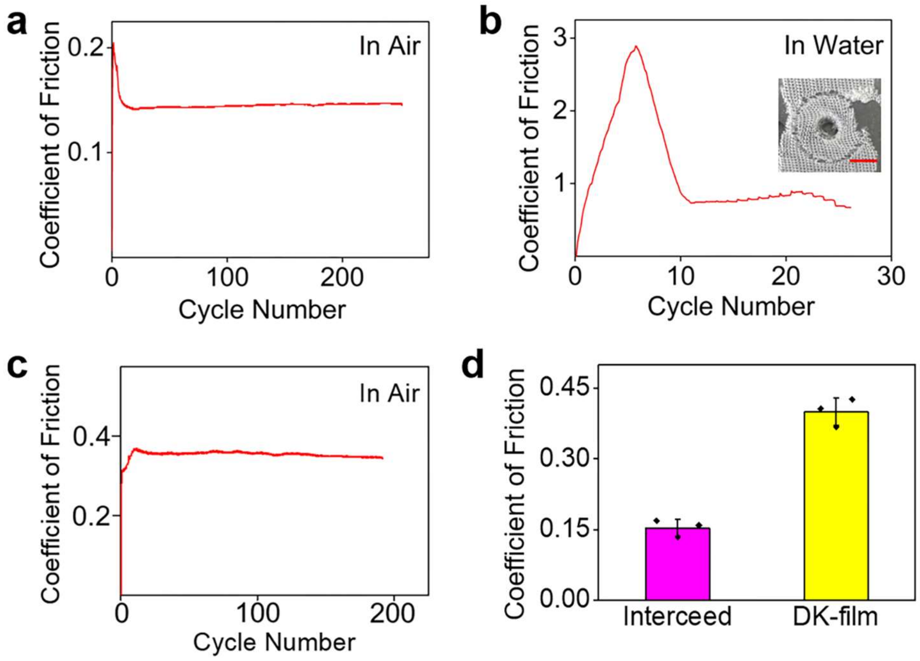

Supplementary Fig.4 The result of the tribological test operated under a rotation mode. **a** COF-testing cycle curve of Interceed in air. **b** COF-testing cycle curve of Interceed in water. Scale bar: 7.5 mm. Note that a larger rotation radius was used in the tribological test to fulfil the experiment under an aqueous condition. **c** COF-testing cycle curve of DK-film in air. **d** Comparison of COF value between Interceed and DK-film. The data in **d** are shown as mean ± SD, and the error bars represent SD (n = 3 independent experiments).

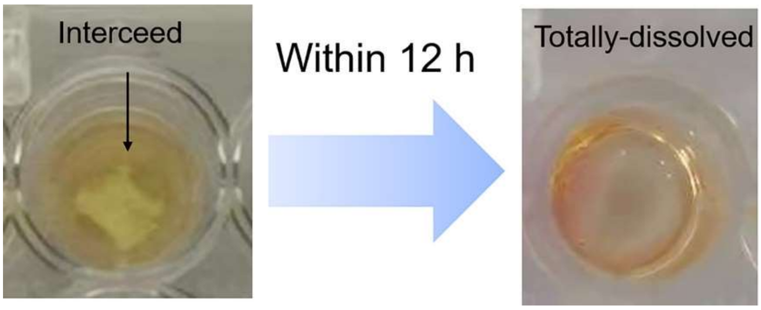

Supplementary Fig.5 Photos of Interceed membrane after being soaked in the culture medium within 12 h.

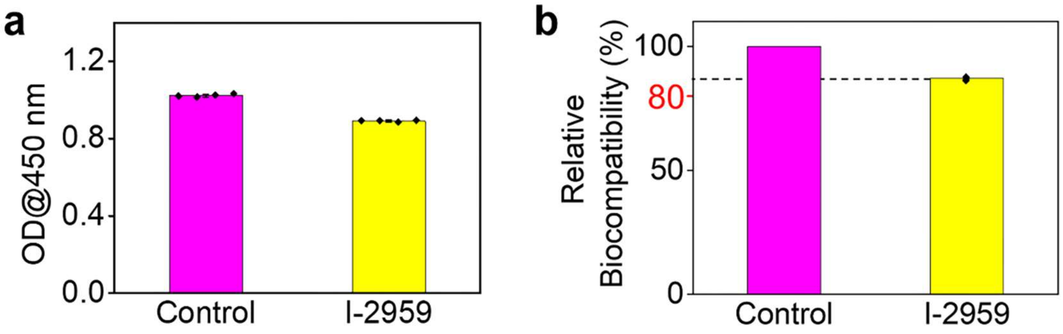

Supplementary Fig.6 The *in vitro* biocompatibility evaluation of I-2959 based on CCK-8 assay by co-culturing with fibroblasts for 24 h. **a** Optical density (OD) values of control and I-2959 groups at 450 nm. **b** Relative biocompatibility calculated from the OD values. The data in **a** and **b** are shown as mean ± SD, and the error bars represent SD (n = 4 independent experiments).

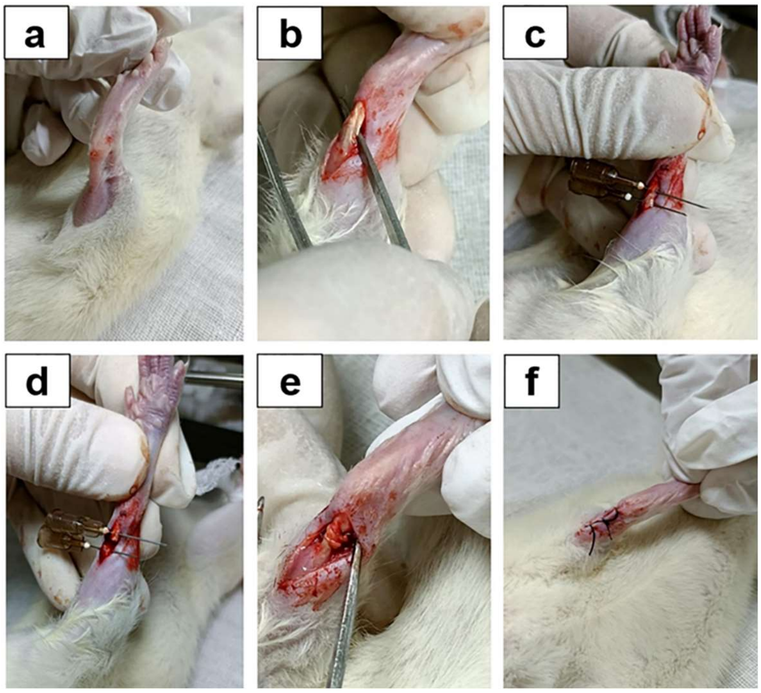

Supplementary Fig.7 Photos showing the typical procedures for establishing rat tendon adhesion model. **a** anesthetization. **b** tendon exposure. **c** pre-immobilization. **d** tendon transection. **e** suturing. **f** skin closure.

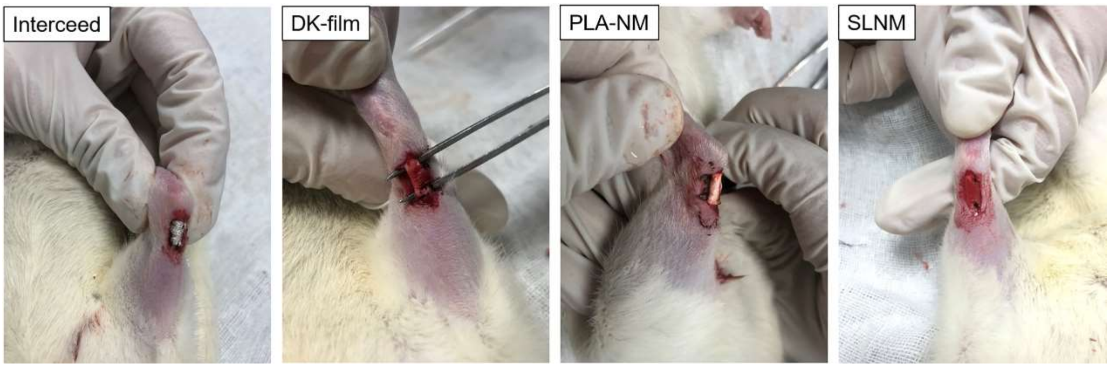

Supplementary Fig.8 Photos showing the implantation of the membrane samples in the Interceed, DK-film, PLA-NM, and SLNM groups before skin closure in the rat tendon adhesion model.

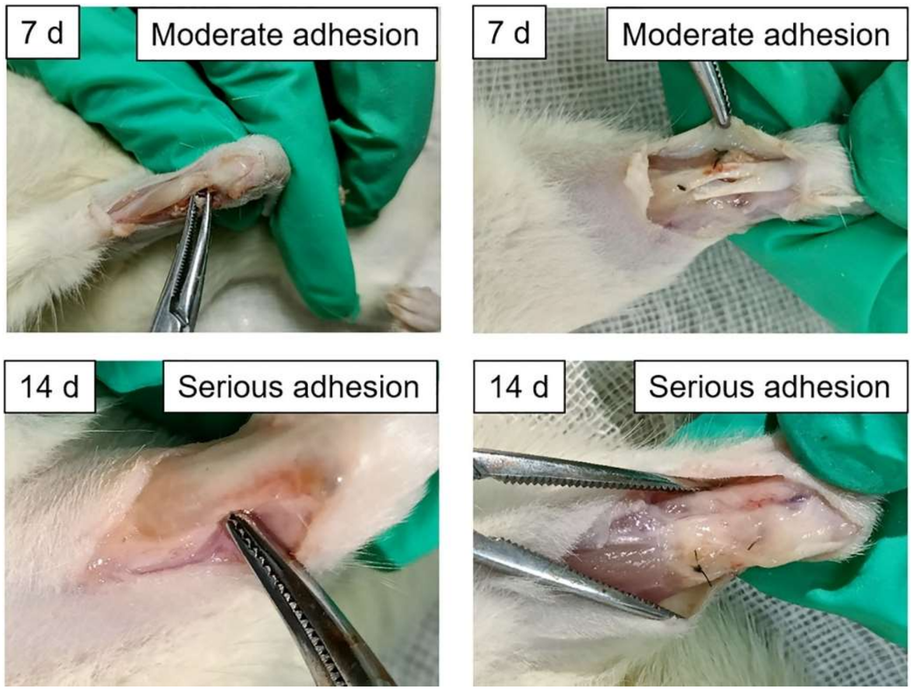

Supplementary Fig.9 A preliminary test for the determination of tissue harvesting time in the rat tendon adhesion model.

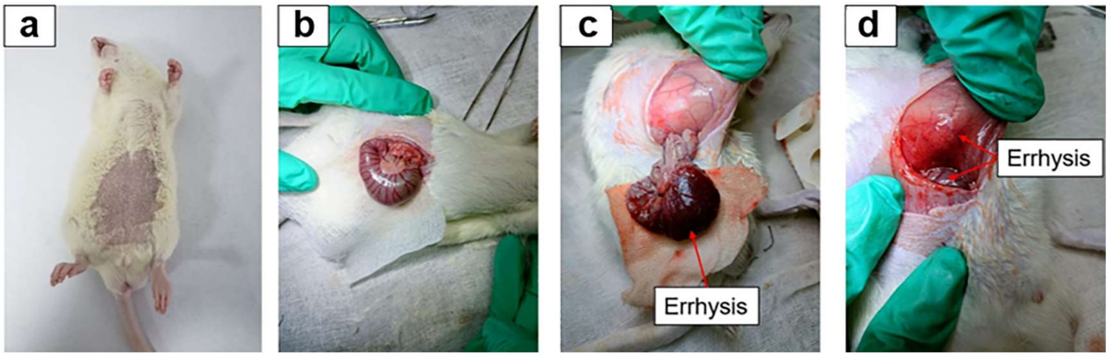

Supplementary Fig.10 Photos showing typical procedures for establishing rat abdominal adhesion model. **a** anesthetization. **b** abdomen incision exposing cecum and opposite abdominal wall. **c** cecum errhysis. **d** abdominal wall errhysis before skin closure.

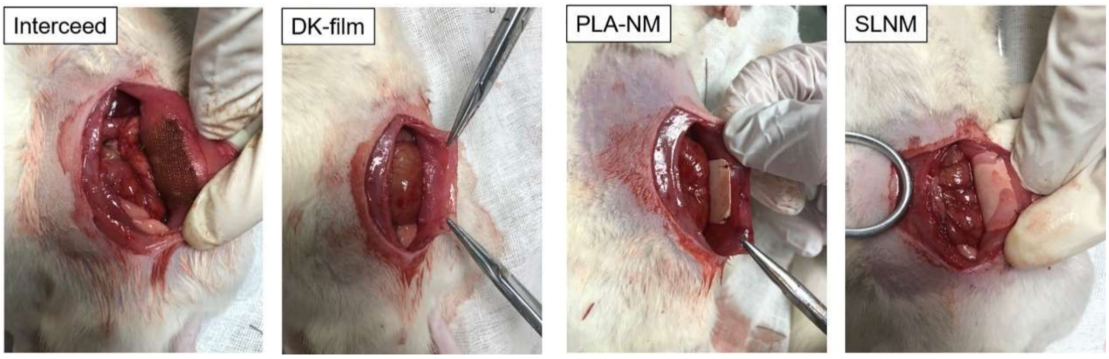

Supplementary Fig.11 Photos showing the implantation of the membrane samples in the Interceed, DK-film, PLA-NM, and SLNM groups before skin closure in the rat abdominal adhesion model. The membranes were sutured on the surface of the abdominal wall.

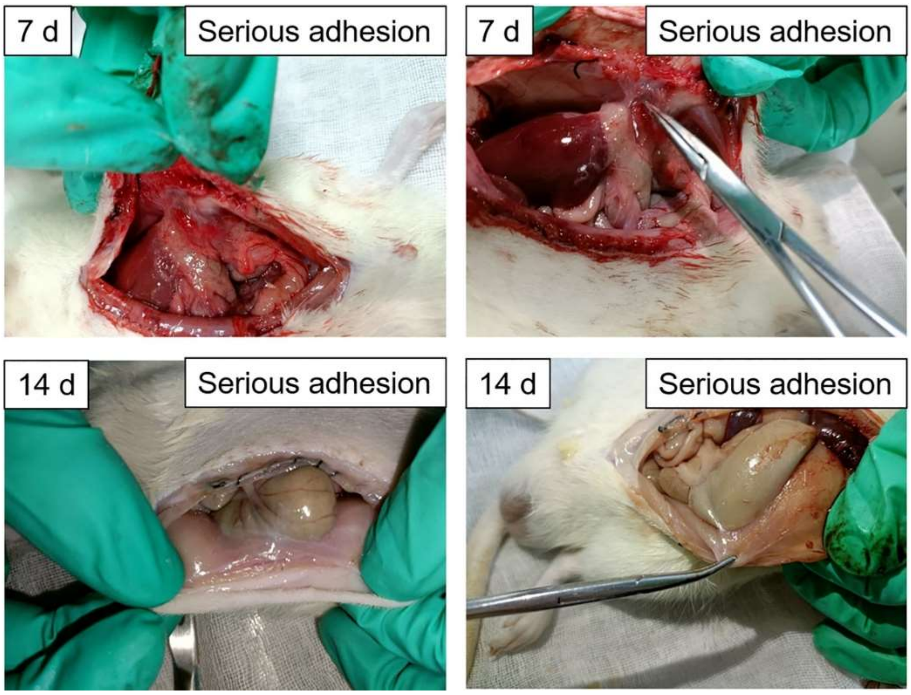

Supplementary Fig.12 A preliminary test for the determination of tissue harvesting time in the rat abdominal adhesion model.

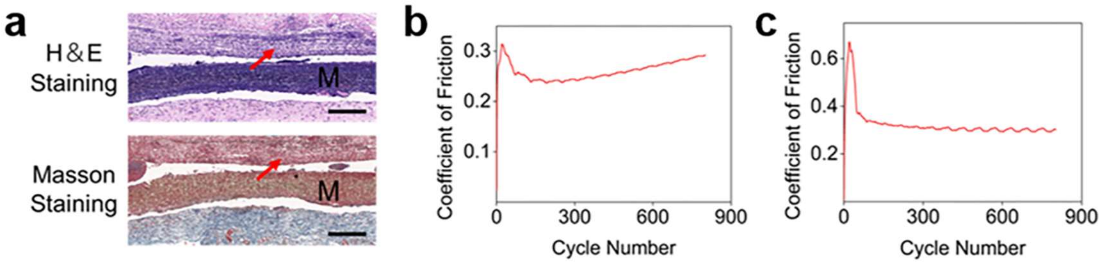

Supplementary Fig.13 The biocompatibility and lubrication properties of nanofibrous membrane modified by hydrogel coating. **a** Representative H&E and Masson staining images. M: Membrane. Scale bar: 200 μm. Red arrow points to the inflammation position. The experiments were replicated three times independently with similar results. **b** COF-testing cycle curve in air. **c** COF-testing cycle curve in water.
