# Supplementary material for: In-situ growth of robust superlubricated nano-skin on electrospun nanofibers for post-operative adhesion prevention
Source: Nat Commun. 2022 Aug 27;13:5056. doi: 10.1038/s41467-022-32804-0 (PMC9420117; doi:10.1038/s41467-022-32804-0)
Supplement: Supplementary file 2 — Reporting Summary [file 41467_2022_32804_MOESM2_ESM.pdf]

nature portfolio

Corresponding author(s): Shujie Cheng, Hongyu Zhang

Last updated by author(s): Jul 23, 2022

Reporting Summary

Nature Portfolio wishes to improve the reproducibility of the work that we publish. This form provides structure for consistency and transparency in reporting. For further information on Nature Portfolio policies, see our Editorial Policies and the Editorial Policy Checklist.

Statistics

For all statistical analyses, confirm that the following items are present in the figure legend, table legend, main text, or Methods section.

n/a	Confirmed	
☐	☒	The exact sample size (n) for each experimental group/condition, given as a discrete number and unit of measurement
☐	☒	A statement on whether measurements were taken from distinct samples or whether the same sample was measured repeatedly
☐	☒	The statistical test(s) used AND whether they are one- or two-sided<br>*Only common tests should be described solely by name; describe more complex techniques in the Methods section.*
☒	☐	A description of all covariates tested
☐	☒	A description of any assumptions or corrections, such as tests of normality and adjustment for multiple comparisons
☐	☒	A full description of the statistical parameters including central tendency (e.g. means) or other basic estimates (e.g. regression coefficient) AND variation (e.g. standard deviation) or associated estimates of uncertainty (e.g. confidence intervals)
☐	☒	For null hypothesis testing, the test statistic (e.g. F, t, r) with confidence intervals, effect sizes, degrees of freedom and P value noted<br>*Give P values as exact values whenever suitable.*
☒	☐	For Bayesian analysis, information on the choice of priors and Markov chain Monte Carlo settings
☒	☐	For hierarchical and complex designs, identification of the appropriate level for tests and full reporting of outcomes
☒	☐	Estimates of effect sizes (e.g. Cohen's d, Pearson's r), indicating how they were calculated

Our web collection on statistics for biologists contains articles on many of the points above.

Software and code

Policy information about availability of computer code

Data collection	Olympus Image Viewer (cellSens standard, version 3.1) (for Olympus BX53F2 microscope), ZEN software (version 2.3) (for LSM-800, ZEISS, Germany), Avantage software (version 5.9918) (for Thermo Fisher ESCALAB 250XI X-ray photoelectron spectroscopy), Thermo Scientific Maps software (version 3.21) (for FEI QUANTA 200 FEG SEM) and Instron Bluehill software (version 3.0) (for Instron 5567, Norwood, MA, USA).
Data analysis	Image J software (version 1.53q) was used for fluorescence intensity quantification. SPSS software (version 19.0) was used for statistical analysis.

For manuscripts utilizing custom algorithms or software that are central to the research but not yet described in published literature, software must be made available to editors and reviewers. We strongly encourage code deposition in a community repository (e.g. GitHub). See the Nature Portfolio guidelines for submitting code & software for further information.

Data

Policy information about availability of data

All manuscripts must include a data availability statement. This statement should provide the following information, where applicable:

- Accession codes, unique identifiers, or web links for publicly available datasets
- A description of any restrictions on data availability
- For clinical datasets or third party data, please ensure that the statement adheres to our policy

The original data of Fig. 1c and Fig. 2g are provided as a Source Data file. Additional data can be obtained from corresponding authors. Because we need to promote the clinical translation of the technology and need a degree of confidentiality. The data that support the findings of this study are available within this paper and or included in the Supplementary Information, and from the corresponding authors upon request. Source data are provided with this paper.

Field-specific reporting

Please select the one below that is the best fit for your research. If you are not sure, read the appropriate sections before making your selection.

- [x] Life sciences
- [ ] Behavioural & social sciences
- [ ] Ecological, evolutionary & environmental sciences

For a reference copy of the document with all sections, see nature.com/documents/nr-reporting-summary-flat.pdf

Life sciences study design

All studies must disclose on these points even when the disclosure is negative.

Sample size

No statistic method was used to predetermine sample size. The sample sizes in our experiments were determined according to literatures. For all the experiments, we followed the accepted common procedures in the similar studying fields. And it has been our routine practice to conduct experiments upon a relatively large but reasonable size. Results repeated at least three times were highly significant and consistent.
1. Scanning electron microscopy analysis, 3 samples each group were used.
2. X-ray photoelectron spectrometer, 3 samples each group were used.
3. Tribological test, 3 samples each group were used.
4. Thermogravimetric analysis, 3 samples each group were used.
5. Water contact angle test, 3 samples each group were used.
6. Tensile test, 3 samples (size: 5 cm × 6 mm) were used for each group.
7. In vitro biocompatibility test, 5 samples each group were used.
8. In vivo biocompatibility test, 3 rats each group were used.
9. In vitro test of anti-cell adhesion, 5 samples each group were used.
10. In vivo anti-tendon adhesion model experiment, 6 rats each group were used.
11. In vivo anti-abdominal adhesion model experiment, 6 rats each group were used.

Data exclusions

No data were excluded from the analyses in most experiments.

Replication

All experiments were repeated independently at least three times with similar results.

Randomization

The rats were randomly assigned to different experimental groups. Cell samples were randomly allocated into multi-well plates.

Blinding

For all the imaging experiments, the investigators were blinded to group allocation during data analysis and collection. For the other experiments, knowledge of experimental group did not affect assessment of the effect, because there were no expected results and all quantification steps were done in an unbiased way.

Reporting for specific materials, systems and methods

We require information from authors about some types of materials, experimental systems and methods used in many studies. Here, indicate whether each material, system or method listed is relevant to your study. If you are not sure if a list item applies to your research, read the appropriate section before selecting a response.

Materials & experimental systems

n/a	Involved in the study	
	x	Antibodies
	x	Eukaryotic cell lines
x		Palaeontology and archaeology
	x	Animals and other organisms
x		Human research participants
x		Clinical data
x		Dual use research of concern

Methods

n/a	Involved in the study	
x		ChIP-seq
x		Flow cytometry
x		MRI-based neuroimaging

Antibodies

Antibodies used

Rabbit Anti-Vinculin Monoclonal Antibody (Abcam, ab129002, Monoclonal, 1:200); Rabbit Anti-TNF alpha Polyclonal Antibody (Bioss, bs-10802R, Polyclonal, 1:200); Rabbit Anti-Collagen III Polyclonal Antibody (Servicebio, GB111629, Polyclonal, 1:500); Goat Anti-Rabbit IgG (H+L) Highly Cross-Adsorbed Secondary Antibody (Invitrogen, A11034; 1:400).

Validation

Rabbit Anti-Vinculin Monoclonal Antibody (Abcam, ab129002) (Citation: Cell Mol Life Sci 78:227-247 (2021), DOI: 10.1007/s00018-020-03485-z)
Rabbit Anti-TNF alpha Polyclonal Antibody (Bioss, bs-10802R) (Citation: Nanoscale, 2019,11, 6693-6709, DOI: 10.1039/c8nr10013f)
Rabbit Anti-Collagen III Polyclonal Antibody (Servicebio, GB111629) (Citation: Front. Mol. Biosci. 8: 690170, DOI: 10.3389/fmolb.2021.690170)

	Goat Anti-Rabbit IgG (H+L) Highly Cross-Adsorbed Secondary Antibody (Invitrogen, A11034, www.thermofisher.cn/cn/zh/antibody/product/Goat-anti-Rabbit-IgG-H-L-Highly-Cross-Adsorbed-Secondary-Antibody-Polyclonal/A-11034)

Eukaryotic cell lines

Policy information about cell lines

Cell line source(s)	NIH/3T3 cells were obtained from NIH Swiss mouse embryo continuous passage cell lines (Cell bank of the Chinese Academy of Science).
Authentication	Cell lines were authenticated by short tandem repeat profiling.
Mycoplasma contamination	All cell lines were tested negative for mycoplasma contamination.
Commonly misidentified lines (See ICLAC register)	There were no commonly misidentified lines in this study.

Animals and other organisms

Policy information about studies involving animals; ARRIVE guidelines recommended for reporting animal research

Laboratory animals	The animals we used in this study were SD rats. Rats were kept with controlled temperature (20-25°C), humidity (40-60%) and light cycle (12 h light/dark). Four-week-old male rats were used in animal experiments.
Wild animals	The study did not involve wild animals.
Field-collected samples	The study did not involve samples collected from field.
Ethics oversight	This study was approved by the Animal Experimental Ethics Committee of Affiliated Hospital of Hebei University (IACUC-2021XS006), and the experiments were conducted in strict accordance with the instructions for the care and use of laboratory animals.

Note that full information on the approval of the study protocol must also be provided in the manuscript.
